# Immunometabolism signature derived from on-treatment tumor specimens predicts immune checkpoint blockade response in metastatic melanoma

**DOI:** 10.1007/s12672-025-02428-z

**Published:** 2025-07-01

**Authors:** Shuzhao Chen, Shutong Liu, Jiangtao Song, Xiaojin Li, Huaze Liao, Shuiqin Chen, Yuanbin Song, Yun Wang, Yang Liang, Qianqian Huang, Weiran Lv

**Affiliations:** 1 Department of Hematology, The First Affiliated Hospital of Sun Yat-Sen University, Guangzhou, 510000 China; 2 Department of Thyroid and Breast Surgery, The First Affiliated Hospital of Shantou University Medical College (SUMC), Shantou, 515000 China; 3 Department of Hematology Oncology, State Key Laboratory of Oncology in South China, Guangdong Provincial Clinical Research Center for Cancer, Sun Yat‐sen University Cancer Center, Collaborative Innovation Center for Cancer Medicine, Guangzhou, 510000 China; 4 Faculty of Environmental Science and Engineering, Kunming University of Science and Technology, 727 Jingming South Road, Kunming, 650500 Yunnan China

**Keywords:** Immunometabolism Signature, Immune checkpoint blockade therapy, On‐treatment tumor specimens, Metastatic melanoma, Biomarker

## Abstract

Gene signatures have been developed to predict the immune checkpoint blockade (ICB) response and prognosis in patients with melanoma. However, most of these signatures are obtained from pre-treatment biopsy samples, and there is no predictive combination of immune and metabolic signatures from patients receiving treatment. In this study, using the Elastic Net Regression (ENLR) algorithm, we built an immunometabolism signature using on-treatment (IMME-ON) tumor specimens based on clinical information and transcriptome data from patients with metastatic melanoma after anti-PD1 and or anti-CTLA4 treatment. The IMME-ON signature was validated in three independent datasets of metastatic melanoma, achieving area under the curve (AUC) values of 0.79–0.86. We also combined all the test samples and obtained an overall AUC of 0.82 for the IMME-ON signature. Based on the IMME-ON signature, subjects were divided into high- and low-scores groups using the mean score. ICB response rates were higher in the high-scoring cohort subjects than the low-scoring subjects. Patient with high scores tended to have better survival outcomes than did those with low scores. In conclusion, we identified and verified an immunometabolism signature that provides a theoretical basis for applying such signatures derived from on-treatment tumor samples to predict therapeutic responses to ICB therapies.

**Supplementary Information:**

The online version contains supplementary material available at 10.1007/s12672-025-02428-z.

## Introduction

Immune checkpoint blockade (ICB) has revolutionized the treatment of advanced melanoma and many other cancer types. However, most patients with cancer do not derive long-term benefits from ICB therapy [1–3]. Robust clinical tools that guide ICB therapies can considerably decrease side effects, drug resistance, and costs. Therefore, identification of robust signatures predictive of responses to ICB therapies is warranted to inform and optimize therapeutic decisions.

Several biomarkers for predicting the response to ICB have been identified in the treatment of metastatic melanoma, such as cytolytic activity [4], aneuploidy [5], deep sequencing of T-cell receptor DNA [6], and tumor mutation burden (TMB) [7]. In addition, functional gene expression signatures, such as the NLRP3 inflammasome [8], immune cell signature [9, 10], inflammatory response signature [11, 12], Tumor-associated endothelial gene signature, glycoprotein VI-mediated platelet activation signaling pathway signature [13] and immune-predictive score (IMPRES) [14], are associated with response to ICB therapy. However, these signatures were evaluated using pre-treatment biopsies.

Metabolic regulation and products are tightly and ubiquitously linked to immune cell activation and functions [15]. A growing body of evidence shows that controlling the metabolic pathways of T cells or cancer cells can overcome T-cell dysfunction and reprogram the metabolic balance in the tumor microenvironment [16]. A recent study has shown that PD-1 signals metabolically promote long-term memory homeostasis by regulating the critical balance between mTOR-dependent anabolic glycolysis and fatty acid oxidation programs [17]. A combination of immune and metabolic signatures may provide a better predictive model for ICB therapy. For example, a recent study developed pathway-related signatures, including lipid metabolism and PD1 signaling, to construct a predictive model tailored to on-treatment samples [18]. Another study revealed that a combination of host immune metabolic biomarkers is valuable for response prediction [19]. However, to date, whether immunometabolism signatures of on-treatment samples can predict patient responses to ICB therapies and outcomes in metastatic melanoma is unknown.

In this study, we obtained transcriptomic and clinical data on the response to ICB on-treatment in metastatic melanoma and developed an immunometabolism signature to predict the response to ICB therapies for on-treatment samples using the elastic net regression (ENLR) algorithm.

## Methods

### Data and resources

Gene expression and associated clinical data from on-treatment tumor samples of four ICB datasets, namely, the Riaz et al. (GEO accession number: GSE120575) [20], Gide et al. (BioProject accession number: PRJEB23709) [21], Lee et al. (EGA accession number EGAD00001005738) [22], and Abril-Rodriguez et al. (dbGaP accession number: phs001919.v1.p1) datasets [23], were analyzed in this study. Samples were excluded from the study based on the following criteria: (1) insufficient response evaluation data and (4) lack of RNA sequencing data. Patient responses to ICB therapy were defined using the RECIST criteria. Responders were defined as patients who achieved either partial response (PR), complete response (CR), or stable disease (SD) with progression-free survival (PFS) longer than 180 days, whereas non-responders were defined as patients with progressive disease (PD) or SD with PFS shorter than 180 days.

### Single-sample gene set enrichment analysis (ssGSEA)

Raw counts of RNA-seq genes were normalized to transcripts per kilobase million (TPM). The single-sample Gene set enrichment analysis (ssGSEA) function of the “GSVA” R package was used to calculate the ssGSEA score for each immune and metabolic gene signature (Supplementary Table S1). The heatmap was used to visualize the ssGSEA values. Each row represented a specific pathway, and the column represented samples.

### Evaluation of signature score

To ensure robust model evaluation, we employed stratified random sampling for data partitioning, implemented through the cv.glmnet function in R. To mitigate potential data leakage risks, we implemented the transcriptomic similarity analysis. We calculated pairwise Pearson correlations between all samples based on their transcriptomic profiles (gene expression data). Over 95% of sample pairs had correlations below 0.9 (Supplementary Figure S1a), a threshold commonly used to define near-identical profiles in transcriptomic studies [24, 25]. Principal Component Analysis (PCA) further confirmed the heterogeneity of our dataset, with tight clustering observed in only a minority of samples (Supplementary Figure S1b). Each patient in the Riaz et al. cohort was represented by a single, unique sample, ensuring no repeated samples from the same patient and eliminating the risk of intra-patient data leakage. Furthermore, an elastic net penalized logistic regression model was implemented on the training dataset using the “glmnet” R package. We tested fold numbers ranging from 3 to 10 and evaluated the predictive performance of our model using the Elastic Net algorithm. Among the various fold numbers tested, the fourfold model achieved the highest average AUC (0.8276) across and lowest variability (Standard Deviation = 0.0260) across three test datasets (Supplementary Table S2). So fourfold cross-validation was performed on the training dataset to test the generalizability of the model and avoid overfitting [26]. Specifically, the dataset was divided with stratification into four subsets, one of which was used in each trial as a test set to evaluate the classifier trained using the rest of the dataset. The cost-sensitive classification approach was used to address the issue of imbalanced class sizes. In addition, a receiver operating characteristic (ROC) curve was plotted by the R software “ROCR” package to calculate the area under the curve (AUC) value of the ROC curve of each prediction model.

### Statistical analysis

The significance of on-treatment differences between responders and non-responders in the ICB cohorts was tested using a two-sided Wilcoxon's rank-sum test. The area under the receiver operator characteristic curve (AUC) was used to assess the prediction accuracy, and the AUC was derived using the R package ROCR. The Coxph function in R was used for multivariate Cox regression analysis. The survival curves were estimated according to the Kaplan–Meier method and compared using the log-rank test. The cutoff point was the mean odds ratio, and samples were separated into a low-signature score group and a high-signature score group. All statistical analyses were carried out using R software version 4.1.0 and Prism Graphpad software (https://www.graphpad.com/scientific-software/prism/).

## Results

### Patient cohorts

**Fig. 1:**
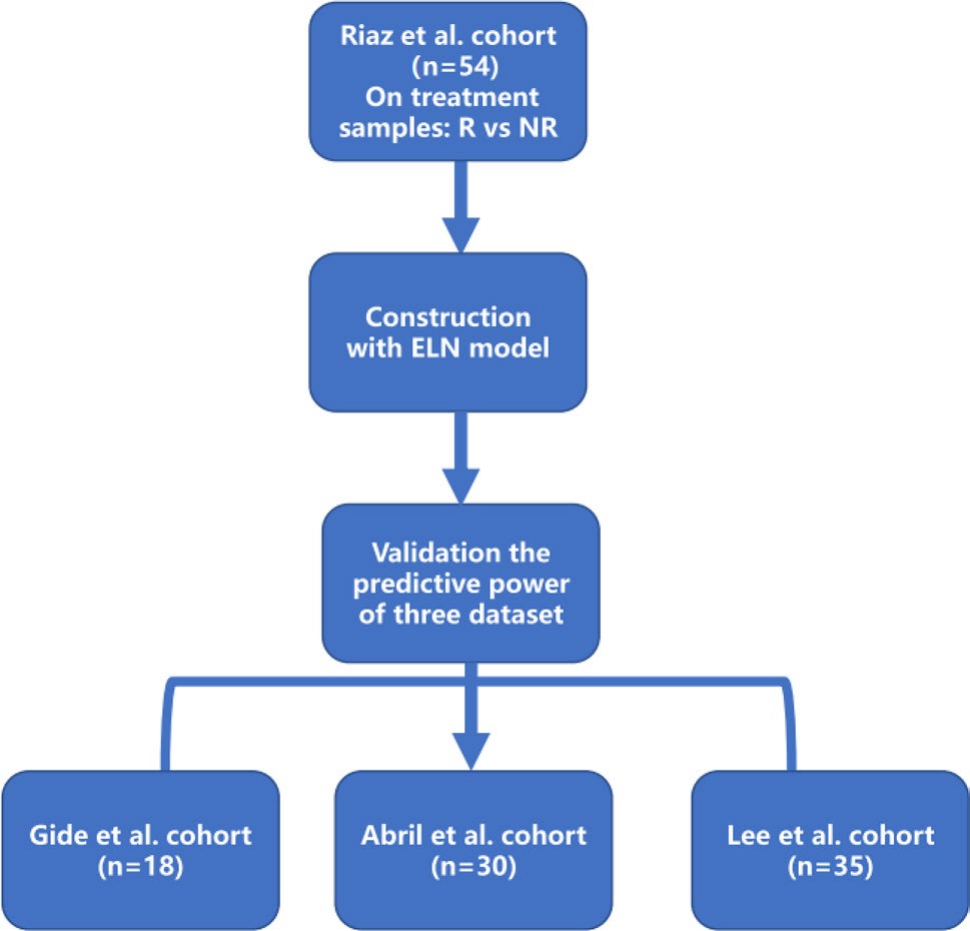
The flow chart of IMME-ON signature score construction

In this study, we used RNA-Seq and clinical data from patients with metastatic melanoma undergoing anti-PD-1 therapy. The Riaz et al. dataset had the largest sample size and was used as the training set for developing the model, while the smaller datasets (Gide et al., Abril-Rodriguez et al., and Lee et al.) were used as validation sets to improve statistical power. The flowchart is shown in Fig. 1.

In the Riaz et al. dataset, a total of 54 biopsies at the timepoint of on-treatment were included in the analysis. 21 biopsies were from R (Responder), and 33 biopsies were from NR (No Responder). All patients received anti-PD1 monotherapy. In the Gide et al. dataset, 17 patients with 18 on-treatment biopsies (11 for R and 7 for NR) received anti-PD1 monotherapy. The median age of the patients was 56 years, and 41% were over 60 years old. Of the patients, 53% were male. In the Lee et al. dataset, 23 patients with 35 on-treatment biopsies (6 for R and 29 for NR) were treated with anti-PD1 monotherapy, including nivolumab (Nivo) or pembrolizumab (Pembro). The Abril-Rodriguez cohort included 31 on-treatment tumor samples (*n* = 5 for R and *n* = 26 for NR). The baseline characteristics of the patients are presented in Supplementary Table S3.

### Establishing the signature score

**Fig. 2:**
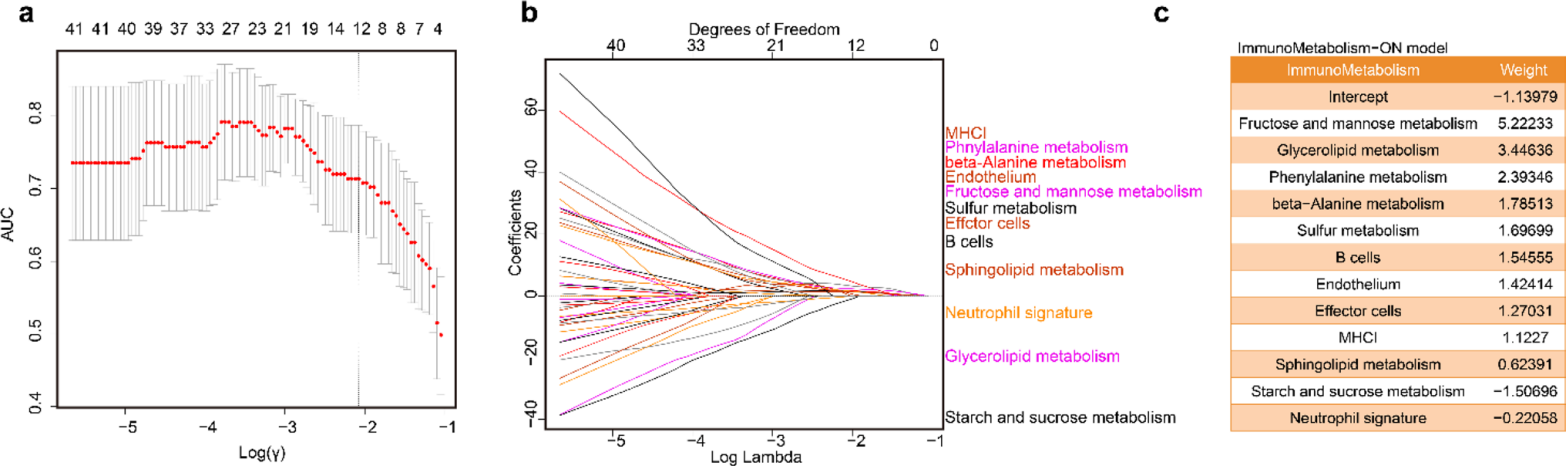
IMME-ON signature for on-treatment samples from Riaz et.al. cohort. **a, b** The modelʹs training parameter selection process was used to generate the Riaz et al. on‐treatment samples to generate the IMME-ON signature. To avoid overfitting, threefold cross‐validation was performed with the parameter setting as ʺtype.measure = auc, family = ‘binomial’.ʺ **c** IMME-ON signatures consisted of twelve selected frames associated with the effect sizes (variable weights) from the elasticnet penalized logistic regression model

We selected 69 immune and metabolic signatures (Supplementary Table S1) derived from previous studies[10] and the KEGG PATHWAY database. First, we calculated pairwise correlations between all samples based on their transcriptomic profiles (gene expression data). We used least absolute shrinkage and selection operator (LASSO) regression to produce a set of coefficients for each immune and metabolic signature in the Fig. 2b training set using the ssGSEA algorithm. We then obtained a regularization parameter, lambda, which ranged from 12 to 27, as shown in Fig. 2a, b, to determine the optimized penalty parameter. Signatures were selected by choosing the LASSO regression coefficients with the largest effective sizes as weights (Fig. 2c). Finally, we obtained a predictive ENLR model with 12 effective hub immunometabolism signatures based on Riaz et al. samples treated with anti-PD-1 therapy. Ten positive signatures were selected: fructose and mannose metabolism, glycerolipid metabolism, phenylalanine metabolism, beta-alanine metabolism, sulfur metabolism, B cells, endothelium, effector cells, MHCI, and sphingolipid metabolism. These positive signatures were related to the response to ICB therapy, which is helpful in ICB treatment. The others were negative signatures, including starch and sucrose metabolism and neutrophil signatures. Starch and sucrose metabolism are two important energy pathways in sugar metabolism and are associated with some preneoplastic lesions. Neutrophil signatures are associated with immune cell infiltration. The signature with the most positive coefficient was fructose and mannose metabolism (value: 5.22233), indicating a strong positive relationship with response to ICB therapies. The most negative signature was starch and sucrose metabolism (value: ˗1.50696). We calculated the weighted average of the ssGSEA scores of these 13 signatures, called the immunometabolism-based signature response score.

### Evaluating the predictive model in the Riaz et al. dataset

**Fig. 3:**
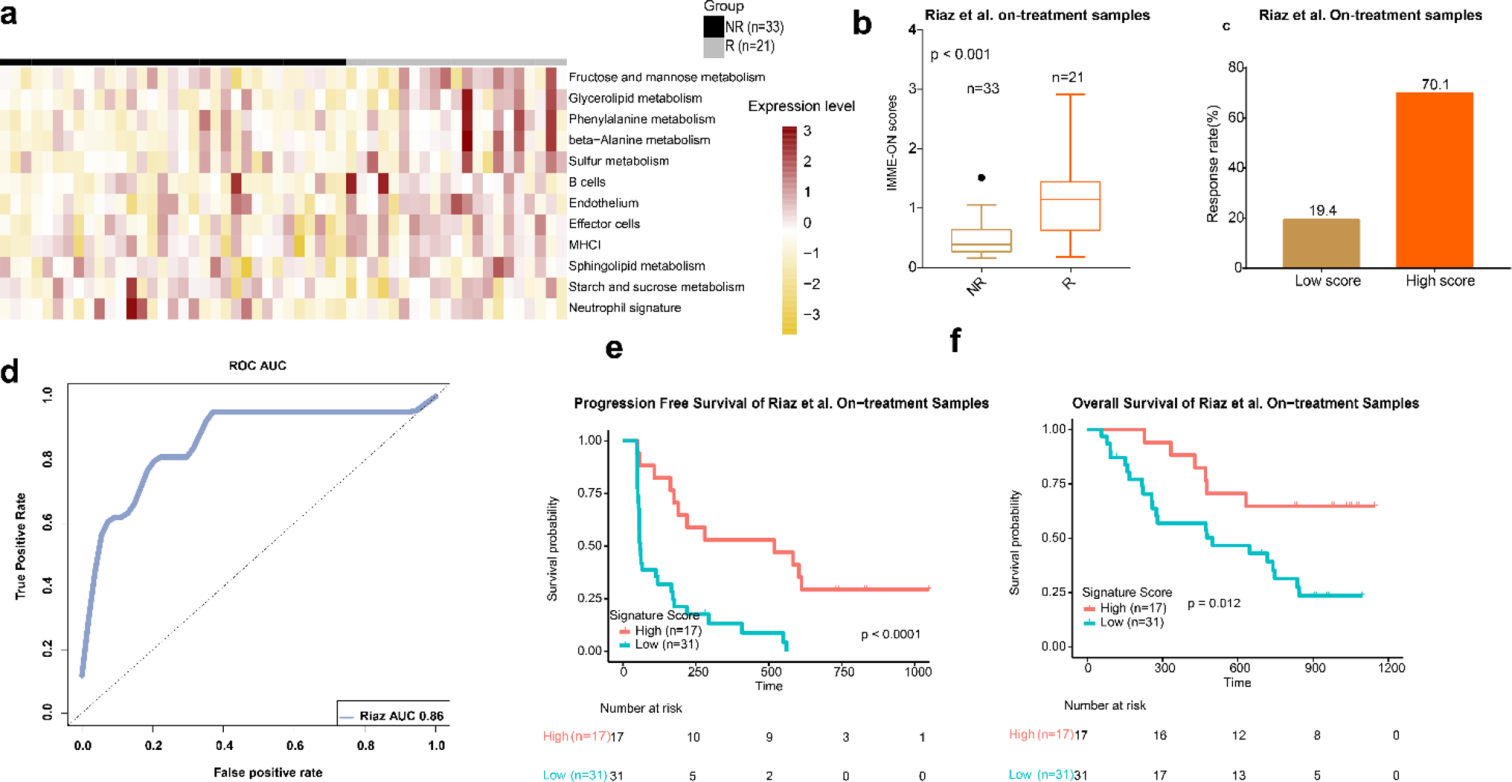
IMME-ON signature for on-treatment samples from Riaz et.al. cohort. **a** Heatmap representing the single sample gene set enrichment analysis value of ontreatment nonresponders (NR) and responders (R) in the Riaz et al. cohort. **b**. Boxplot of IMME-ON signature scores for ontreatment samples from the Riaz et al. datasets. *p* values were computed via a one-sided Wilcoxon rank-sum test. **c** The response rate to immunotherapy in low and high scores groups stratified by IMME-ON signature core in the Riaz et.al. cohort. **d** Receiver operating correlation curve and area under the curve of FGE-PRE signatures for pretreatment samples from the Riaz et al. cohort. **e, f** Kaplan–Meier curves of PFS and OS for pretreatment samples based on IMME-ON signature scores for the Riaz et al. cohort

We selected 54 samples, including 33 non-responders (NR) and 21 responders (R), from the Riaz et al. dataset. We found relatively higher gene expression in the 13 selected signatures for the responders than for the non-responders (Fig. 3a). We also calculated the IMME-ON scores of the on-treatment samples and compared it to the response to ICB in the two groups and found the same difference between the groups (*p* < 0.001; Fig. 3b). Next, we divided the patients into high- and low-scores groups using the mean from the IMME-ON scores. The results suggested that the high-scores group had a higher response rate than did the low-scores group (high-scores,70.1%; low- score, 19.4%; Fig. 3c). The AUC of the ROC curve was 0.86 (Fig. 3d).

To validate the predictive value of the model, Kaplan–Meier (KM) analysis of progression-free (PFS) and overall survival (OS) was performed. The KM curve showed that the high-score group had significantly longer PFS (*p* < 0.0001, Fig. 3e) and OS (*p* = 0.012, Fig. 3f) than did the low-score group.

### Evaluating the predictive model in other datasets

**Fig. 4:**
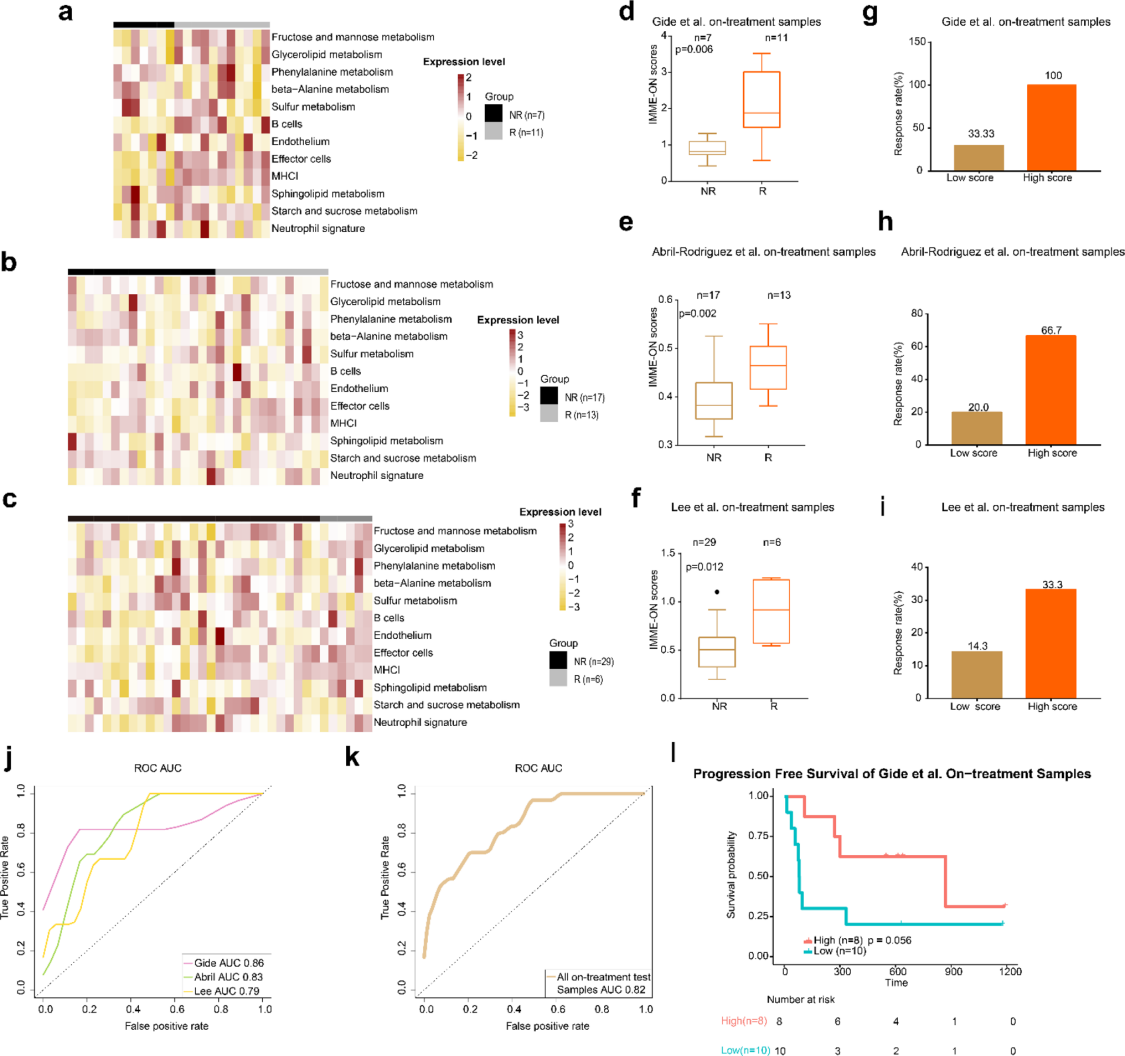
IMME-ON signature for on-treatment samples from validation cohorts. **a–c**. Heatmap representing the single sample gene set enrichment analysis value of on-treatment nonresponders (NR) and responders (R) in the Gide et al., Abril-Rodriguez et al., and Lee et al.cohorts. **d–f**. Boxplot of IMME-ON signature scores for on-treatment samples from Gide et al., Abril-Rodriguez et al., and Lee et al.cohorts. p values were computed via a one-sided Wilcoxon rank-sum test. **g-i**.The response rate to immunotherapy in low and high scores groups stratified by IMME-ON signature core in the Gide et al., Abril-Rodriguez et al., and Lee et al.cohorts. **j** Receiver operating correlation curve and area under the curve of IMME-ON signatures for on-treatment samples from the Gide et al., Abril-Rodriguez et al., and Lee et al.cohorts. **k** Receiver operating correlation curve and area under the curve of IMME-ON signatures for on-treatment samples from all test samples, which combined the Gide et al., Abril-Rodriguez et al., and Lee et al. cohorts. **l** Kaplan–Meier curves of PFS for on-treatment samples based on IMME-ON signature scores for Gide et al. cohorts

To validate the predictive performance of the IMME-ON model, we tested three independent datasets with RNA-seq data available for on-treatment samples, including the Gide et al., Abril-Rodriguez et al., and Lee et al. datasets (Fig. 4a, b, c), and calculated the IMME-ON and odds ratios for all test samples. In the Gide et al., Abril-Rodriguez et al. and Lee et al. datasets, there were extremely significant differences between the NR and R groups (*p* = 0.006, Fig. 4d; *p* = 0.002, Fig. 4e; *p* = 0.012, Fig. 4f).The response rates of the high-risk score group were also higher than those of the low-risk score group (Gide et al., high risk: 100% vs. low risk: 33.33%, Fig. 4g; Abril-Rodriguez et al., 66.7% vs 20.0%, Fig. 4h; Lee et al., high risk: 33.3% vs. low risk: 14.30%,Fig. 4i). We obtained ROC curves and calculated their AUCs (Gide AUC, 0.86; Abril AUC, 0.83; Lee AUC, 0.79; Fig. 4j). Further, we integrated the three datasets and obtained a single AUC for all on-treatment test samples (AUC = 0.82; Fig. 4k). Subsequently, we performed KM analysis of PFS in the Gide et al. on-treatment samples. The result showed that patients with high scores survived longer than those with low scores (*p* = 0.056, Fig. 4l). Although there was no significant difference in the overall survival among Gide et al. (*p* = 0.14, Supplementary Figure S2a). and Lee et al. (*p* = 0.22, Supplementary Figure S2b), the combination cohort showed a certain trend toward significance (*p* = 0.069, Supplementary Figure S2c).

### Evaluating independence of IMME-ON

**Fig. 5:**
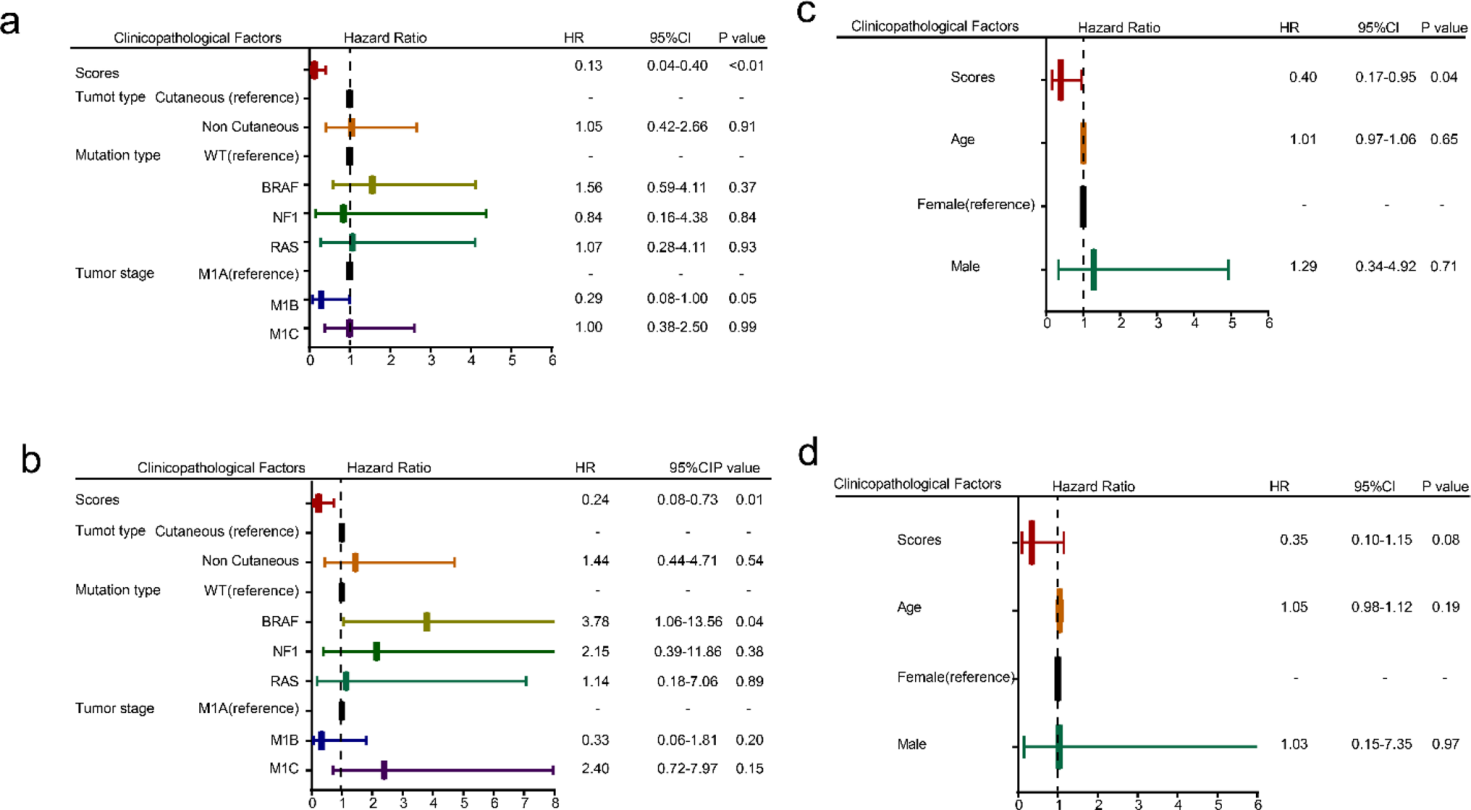
Results of Cox proportional hazards regression for PSS and OS analysis using IMME-ON signature scores for on-treatment samples. **a**, **b** Multivariate Cox regression analysis of progression-free survival (PFS) (**a**) and overall survival (OS) (**b**) in the Riaz et al. cohort, showing the IMME-ON score as an independent prognostic factor for both endpoints after adjusting for clinicopathological variables. **c**, **d** Multivariate Cox regression analysis of PFS (**c**) and OS (**d**) in the Gide et al. cohort, demonstrating that the IMME-ON score was independently associated with PFS but not OS. Hazard ratios (HR) and 95% confidence intervals are shown

We compared these scores with other clinicopathological factors to evaluate their independent prognostic value using multivariate Cox analyses of PFS and OS in the on-treatment samples from the cohort of Riaz et al. The IMME-ON score was identified as an independent prognostic factor for both PFS (Fig. 5a) and OS (Fig. 5b) in the Riaz et al. cohort, after adjusting for additional variables. Multivariate Cox regression analysis revealed that the IMME-ON score was an independent prognostic factor for PFS (Fig. 5c), but not for OS (Fig. 5d) in the Gide et al. cohort. Additionally, we performed the Global Schoenfeld test to evaluate whether the score acted as a time-dependent covariate. All tests showed p-values greater than 0.05 (Supplementary Figure S3a–d, Supplementary Figure S4a–b), suggesting that the assumption of proportional hazards was violated. In summary, the IMME-ON score did not vary over time when predicting OS and PFS, indicating it is not a time-dependent covariate.

We performed a chi-square test to assess the association between the IMME-ON signature and clinical variables, including tumor grade, tumor type (cutaneous vs. non-cutaneous melanoma), mutation type, age, and sex. No statistically significant correlations were found between these variables and the immune-metabolic signature (Supplementary Table S4), indicating that the IMME-ON signature was not significantly influenced by these clinical factors. These findings are limited by the available clinical data, which did not include treatment context, ICB doses, time intervals between diagnosis and sample collection, or disease stage. These factors may potentially affect the signature, but their absence from the dataset prevents further analysis of their impact.

## Discussion

Since the first checkpoint inhibitor, ipilimumab, which targets CTLA-4, gained FDA approval, immunotherapy, especially anti-PD-1 and anti-PD-L1 antibody therapies, has shown inspiring clinical activity in various types of cancers and has become the new standard of anti-cancer therapy [27]. However, a significant subset of patients does not achieve a desirable response to immunotherapy. Although a better understanding of the mechanism underlying tumor-immune interactions is required, TMB, microsatellite instability, and T-cell-inflamed gene expression profiles are critical predictive factors of the response to immunotherapy. Precisely predicting the response of a specific patient to ICB is difficult. Cancer cells exhibiting abnormal metabolism has been well known for nearly a hundred years since the discovery of the Warburg effect [28]. Recent studies have shown that tumor hypoxia, low pH, suppressive metabolites, and low nutrient availability in the TME can hamper the anti-tumor function of T cells in patients receiving T-cell-based immunotherapy. However, the exact mechanisms by which abnormal metabolism in the TME impairs the response to immunotherapy and the key metabolites that determine the outcome of immunotherapy remain largely unknown.

In this study, we developed an immunometabolism-based signature response score based on clinical information and transcriptome data from public datasets of ICB treatment for melanoma. We preliminarily identified 12 immunometabolism signatures using the LASSO regression and ssGSEA algorithms, and then built an ELNR model with these signatures based on on-treatment samples using the Riaz et al. dataset to predict the response in patients with metastatic melanoma and assess the influence of immunometabolism on survival outcomes. We validated the efficacy of IMME-ON using other datasets and demonstrated that IMME-ON could effectively predict the response of patients treated with ICB therapies. Additionally, we tested this hypothesis using multivariate Cox analyses of PFS and OS, strengthening its independent prognostic value.

From the perspective of our study, we emphasize that RNAseq analysis should be performed if on-treatment tumor samples are available. IMME-ON can distinguish clinical patients who are more likely to benefit from ICB therapies. Specifically, a patient with a high IMME-ON score may achieve a better response to ICB therapy than would those with low scores. In contrast, if a patient has a low score, immunotherapy should be administered rationally, combined with other agents such as chemotherapy or radiotherapy, which can remodel the immunosuppressive TME and enhance the anticancer function of tumor-infiltrating immune cells, or switch to other kinds of therapeutic strategies.

Previous studies have provided insights into the response-predictive value of on-treatment samples [13, 29]. We found that the B-cell-related signature is involved in the immune-mediated antitumor response. As part of the immune system, the B-cell signature is a common biomarker of immunotherapy responses [10]. In addition, ICB therapy induces changes in circulating B cells, resulting in high PD1 expression in B cells with low CD21 expression [30]. Many studies have demonstrated the significance of B cells in other tumors [31, 32]. Additionally, we found that the endothelium, effector cells, and MHCI played a positive role in the response to treatment. Recent studies have shown that MHCI is related to immune evasion; however, further research is needed to clarify its role in immunotherapy [33, 34].

Notably, we identified many metabolic signatures that affect response to immunotherapy, including fructose and mannose metabolism, glycerolipid metabolism, phenylalanine metabolism, beta-alanine metabolism, sulfur metabolism, sphingolipid metabolism, and starch and sucrose metabolism. Importantly, fructose and mannose metabolism had the highest weights in the ELNR model. Previous study show that fructose exposure ex vivo promotes elevated cytokine production in mononuclear phagocytes and that a high fructose diet promotes an inflammatory phenotype in vivo [35], which may transform'cold'tumors into'hot'tumors, thus sensitizing anti-PD1 therapy response.

Lipids play important roles in cancer progression and metastasis, thereby influencing patient survival [35–38]. Lipid metabolism also participates in CD1-mediated antigen-presenting by CD1–lipid complexes [39, 40]. Thus, glycerolipid metabolism could affect the immune microenvironment. A recent multi-omics study also noted that glycerolipid metabolism is correlated with the prognosis and immune hallmarks of colon cancer [41]. Beta-alanine metabolism affected barrier function and reduced inflammation in animal experiments, but its detailed role should be further investigated [42]. One possibility is that beta-alanine mainly regulates carnosine synthesis to affect immunity [43]. Carnosine exerts immunoregulatory effects by regulating T cells or macrophages, which can be involved in the ICB response [43–46]. Sulfur metabolism can influence inflammatory responses and immune functions through its major products, including glutathione (GSH), homocysteine (Hcy), and taurine (Tau) [47]. Studies suggest that insufficient or marginal intake of sulfur amino acids may compromise glutathione synthesis and affect the initiation and progression of a large number of anomalies presenting as immune dysfunction [48].

Our study still has some limitations. This study was mainly based on bioinformatic analysis of data extracted from databases and lacks relevant experimental validation. To ensure its applicability in clinical settings, it is essential to further validate the predictive performance of the immunometabolic signature in independent cohorts. Additionally, immunometabolic signature was only validated in cohorts of metastatic melanoma. This study is also limited by the availability of clinical data, which were restricted to tumor grade, tumor type (cutaneous vs. non-cutaneous melanoma), mutation type, age, and sex. Data on treatment context, ICB doses, time intervals between diagnosis and sample collection were not accessible. Additionally, RNA-seq data provides a"snapshot"of the transcriptome at a single time point, and immune infiltrates and metabolic states may fluctuate over time. These factors may influence the interpretation and predictive power of the immune-metabolic signature. As a result, the transcriptomic signatures derived from RNA-seq may not fully reflect the temporal and spatial heterogeneity of these biological processes. This limitation highlights the importance of integrating RNA-seq data with longitudinal or multi-modal datasets, when available, to gain a more comprehensive understanding of these processes.

In conclusion, using the ENLR algorithm, we discovered 12 immunometabolic signatures from the Riaz et al. dataset and constructed an IMME-ON signature score to predict the response of metastatic melanoma to ICB. Patients with high scores tend to benefit more from ICB-based immunotherapy; thus, IMME-ON scores could be helpful in making clinical treatment decisions and predicting the prognosis of metastatic melanoma. The IMME-ON scores could represent a ICB response prediction model with prognostic value in Metastatic Melanoma.

## Supplementary Information

Additional file1

## Data Availability

The data from Riaz et al. used in this study can be accessed in the GEO database under accession code GSE91061. The data from Gide et al. are available in the BioProject database under accession code PRJEB23709. The data from Lee et al. can be found in the EGA database under accession code EGAD00001005738. The data from Abril-Rodriguez et al. are available in the dbGaP database under accession code phs001919.v1.p1. The essential code for model development and the demonstration dataset used in the analyses are available at the GitHub repository (https://github.com/cshuzh/-IMME-ON-signature).
